# Mental health as perceived by Norwegian adolescents living with parental somatic illness: Living in an earthquake zone

**DOI:** 10.1080/17482631.2020.1783064

**Published:** 2020-06-29

**Authors:** Torill Eide, Anne Faugli, Elin Kufås, Nina Helen Mjøsund, Grethe Eilertsen

**Affiliations:** a Department of Health, Social and Welfare Studies, Faculty of Health and Social Sciences, University of South-Eastern Norway, Drammen, Norway; b Division of Mental Health and Addiction, Vestre Viken Hospital Trust, Drammen, Norway; c Department of Nursing and Health Sciences, Faculty of Health and Social Sciences, University of South-Eastern Norway, Drammen, Norway

**Keywords:** Adolescent, children as dependents, health promotion, interpretative phenomenological analysis, mental health, next of kin, parental somatic illness, salutogenesis, “sense of coherence”, stakeholder involvement

## Abstract

**Purpose:**

Severe parental somatic illnesses can influence the entire family, including adolescents’ everyday life, psychosocial functioning and health. Within salutogenesis, it is highlighted that stressor life events, such as parental somatic illness, might lead to a chain of events that can produce tension. There is a lack of in-depth understanding regarding how adolescents living in a situation with a severely somatically ill parent (SIP) perceive their own mental health. The aim of this study was therefore to explore the lived experience of Norwegian adolescents living with an SIP, and their perception of the parental illness’ influence on their mental health.

**Methods:**

Interpretative phenomenological analysis was used. In-depth interviews were conducted with 11 adolescents (aged 13–18 years) who had an SIP. Two adolescents with an SIP participated in study preparation and data analysis.

**Results:**

Adolescents perceived parental somatic illness as a multifaceted influence on their mental health as it represented both personal and relational strain and growth. Their perceptions can be conceptualized by the super-ordinate theme “living in an earthquake zone”, and by two themes, “inner shakes—but not falling apart” and “relational aftershocks—gains and losses”.

**Conclusion:**

For adolescents, parental somatic illness means personal and relational strain and growth.

## Introduction

Severe parental somatic illnesses can influence the adolescents’ family, everyday life and psychosocial functioning (Mauseth & Hjälmhult, 2016; Phillips, 2015; Phillips & Lewis, 2015; Tozer et al., 2019). The term “adolescence”, with its roots in Latin, means “growing to maturity” (P. K. Smith, 2016). In line with person-centredness, we perceive all adolescents as unique, with valuable experiential knowledge regarding their everyday life (Borg & Karlsson, 2017; McCormack & McCance, 2017). In addition, we recognize that developmental issues—such as independence- and identity-seeking—may lead to increased importance of other adolescents during this period (Erikson, 1963; P. K. Smith, 2016).

Health promotion is “the process of enabling people to increase control over, and to improve, their health” (WHO, 1986, p. 1). Salutogenesis is considered to be a relevant theoretical framework for health promotion (Antonovsky, 1996; Vinje et al., 2017), and an essential supplement to pathogenesis (Mittelmark & Bauer, 2017). Antonovsky (1979, 1987) critiqued pathogenesis’ dichotomy between health and illness, seeing health instead as a dynamic movement along an ease–dis/ease continuum. His focus was on understanding what moves persons towards the ease end of this continuum, where the person experience neither pain nor functional limitation, and where health authorities do not identify neither an acute condition, a chronic condition, nor a need for treatment (Antonovsky, 1979). Antonovsky (1985) presented a separate continuum for mental health and defined mental health as the location “of a person on a continuum which ranges from excruciating emotional pain and total psychological malfunctioning at one extreme, to a full, vibrant sense of psychological wellbeing at the other” (Antonovsky, 1985, p. 274). Next, he distinguished between stressors, tension and stress (Antonovsky, 1979, 1987). *Stressors*, which are omnipresent in human life, might create *tension*, which needs to be resolved. Successful resolving might mean a movement towards the ease end of the health continuum and a strengthened sense of coherence, containing the three core components of comprehensibility, manageability and meaningfulness (Antonovsky, 1979, 1987). On the other hand, unresolved tension might be transformed to stress that means a movement towards dis/ease. The decisive factor regarding tension management (the process of dealing with tension and coping with the stressors) is the identification and mobilization of *general resistant resources* (GRRs). A GRR is a characteristic of the individual, primary group, subculture or society that is effective in avoiding or combating stressors (Antonovsky, 1979). Hence, GRRs are resources at our disposal that can facilitate effective tension management, prevent tensions from being transformed into stress and promote a movement towards ease.

Notably, Antonovsky (1987) distinguished between *chronic stressors* and *stressor life events*. The chronic stressor is a life situation, it is generalized and long lasting, and it is characterized by a general resource deficit. Stressor life events, like parental somatic illness, are specifiable in time and space, and they might be followed by *a chain of events* that produce tension (Antonovsky, 1987). To support adolescents with an SIP in identifying, mobilizing and utilizing sufficient resources, a first—and necessary—step is an in-depth understanding of the perceptions of adolescents with an SIP regarding the chain of tension-producing events that might follow parental somatic illness. The in-depth understanding will be useful as it will highlight areas in which sufficient resources need to be identified, mobilized or added, when aiming at promoting the mental health of adolescents with an SIP.

In Norway, universal access to welfare benefits is a well-established principle. The public health-care system provides and finances most of the country’s health services (Statistics Norway, 2019) and Norway’s health expenses per citizen are among the highest in Europe (Statistics Norway, 2018). Norwegian health personnel are obliged to provide information to and necessary follow-up of children and adolescents with ill parents (Helsepersonelloven, 1999), but there are still indications that health personnel lack competence concerning the influences of parental illnesses on children (Helsedirektoratet, 2015). One reason could be the shortage of studies from the Nordic countries, as most qualitative studies (from between 2008 and 2019) with data from adolescents (13–18 years of age) with an SIP on their experiences of everyday life are from the UK, the USA and Australia. Those studies highlight how parental somatic illness means a fundamental life change for the adolescents, and experiences of worry, sadness and a lack of control (Bogosian et al., 2011; Clemmens, 2009; Kennedy & Lloyd-Williams, 2009a; Phillips, 2015; Phillips & Lewis, 2015; Tozer et al., 2019). The adolescents report that their relationships with their parents change because the ill parent becomes less available (Davey et al., 2011; Phillips, 2015) or because of a redefinition of family roles (Finch & Gibson, 2009; Phillips & Lewis, 2015). Parental somatic illness potentially leads to increased conflict among siblings, more time spent with the extended family (Tozer et al., 2019) and a closer relationship with the ill parent (Turpin et al., 2008). Both parents and the adolescents worry about not having enough time together (Sheehan & Draucker, 2011). Adolescents with an SIP also describe increased domestic responsibilities (Davey et al., 2011), which might limit their freedom and time spent with friends (Bogosian et al., 2011; Kennedy & Lloyd-Williams, 2009a; Phillips, 2015). Spending less time with friends due to practicalities such as a lack of transport (Bogosian et al., 2011), and because their friends found it difficult to be confronted with the illness, have also been reported (Kennedy & Lloyd-Williams, 2009a). Finally, some studies highlight positive experiences of post-traumatic growth (Bogosian et al., 2011; Davey et al., 2011; Phillips & Lewis, 2015), including new values, enhanced relationships (Kennedy & Lloyd-Williams, 2009a; Phillips, 2015; Tozer et al., 2019), a greater appreciation for life and an increased sense of personal strengths (Kissil et al., 2010).

From recent qualitative studies, we have identified only two Swedish and two Norwegian studies that report on the experiences of everyday life of adolescents with an SIP. Below, we highlight results that complement those presented above. Norwegian adolescents experiencing parental multiple sclerosis describe an unpredictable and uncertain everyday life, and their struggle to preserve control (Mauseth & Hjälmhult, 2016). They worry about finances, and they feel responsible for keeping the ill parent in a good mood. Swedish adolescents experiencing parental multiple sclerosis describe never being able to forget about the illness, and feelings of being obliged to help at home (Boström & Nilsagård, 2016). Swedish adolescents experiencing parental cancer describe everyday life as a new, often frightening situation (Jansson & Anderzen-Carlsson, 2017). They found it deeply troubling to witness their parent’s pain and sadness, and were disappointed if they could not realize their own ideals of being independent, unemotional, caring and positive. They often kept the parent’s illness private in order to preserve friendships and to avoid personal questions; moreover, they described how friends did not support them in the way they had anticipated (Jansson & Anderzen-Carlsson, 2017). Norwegian adolescents (aged 16–25 years) with a parent with cancer, a mental illness or a substance abuse problem described experiences of stigma and pitying; a wish to be treated like ordinary adolescents; and difficulties balancing their own needs and the burdens and boundaries created by parental illness (Gullbra et al., 2016). Additionally, experiences of life as unpredictable and feelings of guilt were especially apparent among participants with parents with mental illness or substance abuse problems but were also seen among participants experiencing parental cancer.

Further, the qualitative studies on adolescents with an SIP focus on two more significant themes. One deals with adjustment and coping (Bogosian et al., 2011; Boström & Nilsagård, 2016; Davey et al., 2011; Kennedy & Lloyd-Williams, 2009a; Phillips, 2015), including post-traumatic growth (Kissil et al., 2010) and perceptions of what supports coping (Maynard et al., 2013). Another significant theme, information and communication, includes the views of adolescents with an SIP regarding information (Jansson & Anderzen-Carlsson, 2017), information needs (Kennedy & Lloyd-Williams, 2009b) and how to be informed about their parent’s disorder (Thastum et al., 2008). Moreover, it includes studies that focus on adolescents’ experiences of learning about (Finch & Gibson, 2009) and understanding their parent’s diagnosis and treatment (Huang et al., 2018), and their experiences and patterns of communication in the family (Clemmens, 2009; Forrest et al., 2009).

The studies mentioned above contribute important knowledge regarding adolescents’ experiences of living with a somatically ill parent. However, there remains a paucity of studies framed by salutogenesis, and studies that examine, in depth, the lived experience of adolescents with an SIP, especially how parental illness influences their mental health. In-depth knowledge of the lived experience of Norwegian adolescents with an SIP would be valuable for health personnel when providing information to and necessary follow-up of those adolescents. The aim of the study was to explore in depth the lived experience of Norwegian adolescents living with an SIP and their perception of how parental illness influenced their mental health.

## Methodology

In this study, an interpretative phenomenological analysis (IPA) methodology was applied (J. A. Smith et al., 2009). IPA builds on the work of Husserl, a phenomenologist who argued for a systematic exploration of everyday lives (J. A. Smith et al., 2009). One hermeneutical insight that informs IPA is Heidegger’s questioning as to whether human beings can truly know without interpretation. Accordingly, the interpretative role in IPA has emphasized the researcher is inevitably active when “trying to make sense of the participant trying to make sense of what is happening to them” (J. A. Smith et al., 2009, p. 3). Ideography informs IPA through its focus on the particular, building on the assumption that individual stories have the power to bring us closer to significant aspects of the general. IPA researchers aim at adopting an insider perspective of the phenomenon in question, while emphasizing that researchers access the *meaning* people ascribe to their experience, rather than the experience itself (J. A. Smith et al., 2009).

### Adolescent co-researchers

There is a growing focus on involving end users in research concerning the end users themselves (Wright & Kongats, 2018), and the inclusion of co-researchers with experiential knowledge on the phenomenon in question is argued to increase the quality of IPA research (Mjøsund et al., 2017). In order to strengthen the study’s insider perspective, two adolescents with an SIP (and not study participants) contributed as co-researchers from the study’s preparation period through the research process, including the dissemination of preliminary results at an international conference (www.ittakesavillage2019.com). The adolescent co-researchers brought their experiential knowledge (Borg & Karlsson, 2017; McCormack & McCance, 2017) to the study, and efforts were made to facilitate opportunities for them to voice those experiences. For example, sessions in which two of the authors and both adolescent co-researchers participated were organized such that the adolescent co-researchers had time for individual reflections before conversations in plenary. In each plenary discussions, the adolescent co-researchers were invited to present their reflections first. All tasks were voluntary, and the adolescent co-researchers had a standard employment contract. The adolescent co-researchers’ specific contributions are presented below.

### Sample, participants and setting

Seeking an understanding of the lived experience of adolescents with an SIP requires obtaining data from the adolescents themselves. The sample was purposively selected, small and relatively homogeneous, as IPA research is concerned with a detailed understanding of particular phenomena in particular contexts (J. A. Smith et al., 2009). The inclusion criteria were i) currently living with a somatically ill parent, and having done so for at least one year; ii) being willing and able to elaborate on their experiences; and iii) being between 13 and 18 years of age. As the study supplements a Norwegian multi-centre study that included families with parents from departments of cancer and neurology (Helsedirektoratet, 2015), adolescents with a parent with either cancer or a neurological disorder are included. The adolescent co-researchers provided input for the writing of the informational material, to ensure the use of age-appropriate language.

Health-care personnel in services for cancer and neurology in three Norwegian municipalities and one hospital trust assisted us by informing patients (parents) with adolescents about the study. These parents then informed their adolescent. If the adolescent wanted to participate, the parent or the adolescent contacted the first author directly to arrange the interview. This recruitment strategy resulted in nine participants. In addition, interest groups for persons with somatic illnesses distributed information about the study to members and their families; this resulted in two additional participants, who contacted the first author directly. Adolescents participated with their informed consent, and with written consent from parents.

The final sample comprised six girls and five boys, who had lived with parental illness in periods lasting from 1 to 16 years (i.e., since birth) (see Table I). The participants’ decisions regarding where to meet resulted in six interviews in hospital offices, four in library study rooms and one in the participant’s home.

**Table I.** Characteristics of participants.

Gender	Age	Ill parent	Parent’s disorder
Boy	13	Father	Neurological disorder*
Girl	13	Father	Cancer
Boy	14	Mother	Cancer
Boy	14	Mother	Neurological disorder*
Girl	14	Father	Cancer
Girl	16	Mother	Neurological disorder*
Girl	16	Mother and father	Cancer and cancer
Boy	17	Mother	Cancer
Girl	17	Mother	Cancer
Girl	17	Mother	Cancer
Boy	18	Father	Cancer

*Neurological disorders included multiple sclerosis, amyotrophic lateral sclerosis and cluster headache.

### Data generation—qualitative interviews

Phenomenology’s focus on a detailed exploration of the phenomenon of interest and the focus of ideography on each participant informed both the planning and conducting of the interviews. To obtain detailed accounts, data were generated through semi-structured, in-depth interviews that also invited open dialogue. An interview guide was developed based on the aim of the study and earlier research. The adolescent co-researchers provided input on the interview guide to ensure that the questions were age-appropriate. Topics centred on the experiences of good mental health and experiences related to the promotion of the participants’ mental health. These topics were explored through questions like, “Could you tell me about a day when you felt good?”; “What do you do to feel good?”; and “What can others do to make you feel good?” The participants were invited to address these broad topics in their own words, with follow-up questions from the interviewer (for example, “Could you describe this even more thoroughly?”) encouraging in-depth descriptions. Before each interview, the interviewer (the first author) memorized the central questions of the interview guide, which were then used flexibly to facilitate participants’ telling of their story. To achieve an inductive-informed feed-forward process, preliminary results from case analysis were included in later interviews, for further exploration. The adolescent co-researchers, who read anonymized transcripts of the first six interviews, participated in identifying such preliminary results—one of which was “parental illness as challenging for friendships”. The length of the interviews was between 48 and 108 minutes (78 minutes, on average) and they were audio-recorded. The interviews were transcribed verbatim, six by the first author and five by a professional.

### Pre-understanding and reflexivity

The literature review informed the authors’ pre-understanding that, from a hermeneutic perspective, is a prerequisite for new understanding (Alvesson & Sköldberg, 2009). These pre-understanding were challenged during the course of the study. For example, the interviewer assessed the first interview as somewhat unsuccessful, as the participant had not elaborated on feelings like sorrow or fear. Retrospectively, this judgement was understood as related to the hermeneutic concept of fore-structures. The interviewers’ pre-understanding held an assumption that adolescents with an SIP suffer; following Heidegger and Gadamer (J. A. Smith et al., 2009), this fore-structure was recognized only after engaging with the interviews analytically. In line with J. A. Smith et al. (2009), we value this retrospective understanding.

### Ethics

The interviewer began each interview by providing written and oral information about the study. Next, the interviewer re-stated that participation was voluntary and that participants could withdraw from the study, to give them a chance to reconsider their participation without potential parental pressure. In addition, the interviewer was highly conscious of respecting any verbal and non-verbal cues that might indicate participants’ unwillingness to share parts of their story.

As adolescents with an SIP are perceived to be in a vulnerable situation, the interviewer called each participant approximately three days following the interview to offer them professional follow-up support if needed. One participant accepted the offer and had one session with a child psychiatrist. As parents of all the participants knew about their child’s participation, and as many participants stated that they spoke extensively to their parents about their life situation, the risk of parents recognizing quotes was determined to be high. Accordingly, precautions to safeguard the anonymity of participants were put into place. First, neither the biographical information in Table I nor pseudonyms are linked to the quotes. Secondly, parental diagnosis is not linked to the biographical information of the participant, as some of the neurological disorders are relatively rare. Finally, quotes were carefully reformulated if their content risked revealing the identity of the participant. Data were stored according to the requirements of the Norwegian Centre for Research Data, which approved the study (project number 51505, January 2017).

### Data analysis

Analysis was conducted according to IPA, which contains two phases (J. A. Smith et al., 2009). The first consists of the analysis of single interviews; the principle of working with each interview separately reflects IPA’s commitment to ideography (J. A. Smith et al., 2009). The second phase consists of analysis across the interviews.

During the first phase, four steps were completed. The first of these was *reading and re-reading* each transcript to become immersed in the data and the perspective of each participant. The second was an *initial noting* of anything of interest on three levels: i) descriptive comments, which were close to the participant’s explicit meaning and thus had a phenomenological focus; ii) linguistic comments, for example, the use of metaphors and expressions of emotion; and iii) conceptual comments, which were interpretative and often took an interrogative form aiming at stimulating further analysis. The third step in the first phase was *developing emergent themes* and involved an analytic shift, working with the initial notes and turning these into emergent themes. Step four, *searching for connections across emergent themes*, involved identifying sub-themes and super-ordinate themes based on content common across the emergent themes.

During phase two, which involved *looking for patterns across cases*, we developed sub-themes, themes and a super-ordinate theme via a repeated process of splitting, merging and re-naming the results from phase one. This process illustrates how the concept of the hermeneutic spiral informs IPA, in a continuous shift between pre-understanding and new understandings, parts and wholes, the text and the interpreter. We aimed at securing internal consistency; ensuring the uniqueness and distinctiveness of the super-ordinate theme, themes and sub-themes; and highlighting commonalities, variations and nuances. Next, we strived for a logical and congruent degree of interpretation across each level of the analysis. The process of analysis was managed with the aid of NVivo, version 12 (QSR International, 2018). The adolescent co-researchers face-validated results, emphasizing their communicative value. Table II illustrates the analysis of participants’ quotes informed the emergent themes and the sub-themes; and the sub-themes that make up the themes.

**Table II.** Examples illustrating the analysis from quotes and descriptive, linguistic and conceptual comments shaping emergent themes, sub-themes and themes.

Quotes	Comments	Emergent themes	Sub-themes	Themes
“*If I explain it, it sounds like our situation is terrible (…) It sounds like I have the worst possible life. However, I do not. I have a good life. Even if it is hard that my father is ill, I feel happy most of the time, I am not sad, and I don’t see myself as pitiful*”.	*Descriptive comment*: Listening to her explaining her situation makes people assume she has the worst possible life. For her, it is not like that. *Linguistic comment*: She contrasts what people assume about her life with how she actually feels, repeatedly, and in different ways: she does NOT have the worst possible life, she is NOT sad, she does NOT perceive herself as pitiful—MOST of the time, she is happy. *Conceptual comments*: When she explains her situation, people assume “automatically” that she is suffering. It is as if she experiences that the only possible conclusion regarding her story is that her life is terrible. That is not the case. How is it for her to think about this? It seems like it really bothers her.	Having an ill parent does not necessarily mean suffering. Still, it is hard, and this “paradox”—feeling happy despite having an ill parent—is hard to explain.	An occupied mind	Inner shakes—but not falling apart
(Participant explains friends’ surprised reactions each time she updates them on her ill mothers’ situation:) “Seriously!!? Are you okay????!”	*Descriptive comment*: Friends react with surprise when being updated on participant’s situation. *Linguistic comment*: Participant’s imitation of friends’ response emphasizes shock, that it is sensational and dramatic. Participant clearly found the reaction uncomfortable. *Conceptual comment*: Drama, horror and the sensational among friends is experienced as an unnecessary, uncomfortable reaction. Does participant lose hope because of the response?	Dramatic reaction when friends learn more	Challenging times for friendships	Relational aftershocks—gains and losses

All authors reflected on the super-ordinate theme, sub-themes and themes, and discussed these until consensus was reached. While principles for assessing quality in IPA research have been described (J. A. Smith et al., 2009), there is no explicit prescription for how to ensure analytical trustworthiness within IPA (Rodham et al., 2015). Thus, for transparency, a thorough description of how we carried out the process of securing analytical trustworthiness follows. First, the first author holistically presented the preliminary sub-themes and themes, and quotes and comments that underpinned her interpretations. Next, when co-authors saw contrasting or complementary ways of understanding the quotes or presenting the results, we reflected on the alternatives—a discussion that sometimes resulted in a rearranging of the sub-themes and themes. For example, quotes that the first author initially interpreted as illustrating relational consequences of parental illness were re-interpreted as *worries* about relational consequences, and were thus relevant to the sub-theme “*an occupied mind”*. This process of assessment, via thorough and critical cooperation and a comprehensive review of preliminary results, reduced the risk that results would simply reflect the pre-understanding of the first author—and thus increased analytical trustworthiness.

## Results

The analysis resulted in one super-ordinate theme interpreted and conceptualized as *living in an earthquake zone*, which in turn features two themes: i) *inner shakes—but not falling apart*; and ii) *relational aftershocks—gains and losses*. These themes consist of four sub-themes (see Table III).

**Table III.** Sub-themes, themes and super-ordinate theme.

Sub-themes	Themes	Super-ordinate theme
An occupied mind Shivering*—*a genesis of personal development	Inner shakes*—*but not falling apart	Living in an earthquake zone
Welded family ties*—*and formation of cracks Challenging times for friendships	Relational aftershocks*—*gains and losses	

The symbol (…) in quotes means that words have been removed from the original transcript for ease of reading.

### Living in an earthquake zone

*Living in an earthquake zone* reflects the participants’ perception of the influence of parental illness on their mental health. First, the metaphor of an earthquake captures their experiences of the onset of the parent’s illness. The unexpected upheaval of having a severely somatically ill parent was described as a dramatic and scary life event: “It’s like a bang, kind of, getting sick. Because it happens so suddenly”. Second, the metaphor captures the meaning participants ascribed to parental illness*—*a repeated, though partly unpredictable, mental strain in everyday life*—*as living in unstable terrain. Just as natural earthquake zones have calm periods as well as active ones, the adolescents’ descriptions revealed changes in how they were influenced by the parental illness. In this study, the “calm” periods were periods when the mental strain was described as being absent. The descriptions also indicated that the duration of the parental illness was important to how they experienced their situation. In a way, it seemed as though they had become habituated to the unpredictable situation.

The participants described the parental somatic illness as creating mental strain—or tension, following Antonovsky (1979, 1987)—along two different dimensions. The first reflects a personal strain, conceptualized as *inner shakes—but not falling apart*, and the second deals with a relational strain, conceptualized as *relational aftershocks*. Though the two dimensions are different, they also overlap and highlight the complexity of the participants’ descriptions. Below, these themes are explored in depth.

### Inner shakes—but not falling apart

Participants described a mind occupied with troubled thoughts and feelings about the parental illness, emotional and cognitive reactions, and ethical dilemmas. We conceptualized this mental strain as “inner shakes”. Still, some participants emphasized that the parental illness had not severely affected their mental health, and others described a valued shift in their personal development. Hence, the mental strain did not necessarily mean “falling apart”.

#### An occupied mind

Participants described having a mind occupied with thoughts about the parental illness, and accompanying feelings. One girl, whose parent had a terminal, incurable condition, explained how she experienced the situation before she, with support from her family and professional follow-up, accepted the situation: “I was sad, almost constantly, and I thought about it all the time. If I only heard the word ‘cancer’, I cried”. As the word itself was hard to avoid and was a reminder, it contributed to repeated mental strain.

One participant began crying when the interviewer asked her to describe an ordinary day, saying, “It is always like this, when I think about it.” There was thus a pattern in her frequent episodes of crying—they occurred each time she thought about her mother’s illness. Contrastingly, another girl expressed the unexpectedness of becoming sad during the interview. She said, “I get tearful now, thinking about it”; her next comment—“O, my God, this happened very suddenly”—indicates that she had not expected the tears.

Participants further described being occupied with understanding their own emotional and cognitive reactions. One girl found it strange that she cried without feeling sad, and that she repeatedly forgot how physically reduced her ill mother was. She described that she had asked her mother for practical help, forgetting how difficult it would be for her mother to provide such help: “I don’t understand, how I could forget it? (…) I think it is really strange. It is a big thing. It is really strange to forget about it.” Her verbal repetitions emphasized how difficult it was for her to make sense of her failure to remember.

Some of the participants were struggling with ethical dilemmas. They wondered if they should talk to friends about their situation, as they felt such conversations would place burdens on their friends. One boy explained that he felt obliged to spend, and enjoy, sunny days outside, as his physically reduced mother was unable to do so. In addition, he felt he could not complain: “I feel that I can’t complain about trivialities (…) It would be like complaining about not liking the food you get at home, to children who don’t get food. It would be wrong, kind of.” He elaborated on why he judged complaining as “wrong”: “I feel selfish for thinking that I have it bad, because she’s the one in a bad situation.” A common trait across the dilemmas seems to centre around when to prioritize one’s needs, or express one’s experiences, “at the expense” of others’ needs and perspectives.

A shared aspect across the participants’ accounts was that the parental illness did not severely influence their mental health. Some reported that they felt good most days, and one boy, who described his family as supportive, stated, “I have not noticed any difference, regarding my mental health, before and after the onset of the illness.” One girl seemed surprised that she felt better as her father’s fatal disease grew more serious. She emphasized that this sounded “strange” and “weird”, words that indicate that she found it challenging to express the mismatch. She continued to emphasize the discrepancy between how the story sounded and how she felt:

If I explain it, it sounds like our situation is terrible (…) It sounds like I have the worst possible life. However, I do not. I have a good life. Even if it is hard that my father is ill, I feel happy most of the time, I am not sad, and I don’t see myself as pitiful.

This participant’s mind seemed occupied with understanding the intuitive paradox of feeling good despite the suffering of a loved one.

#### Shivering—a genesis of personal development

The participants described how parental illness could mean a shift in personal development. To one participant, it meant being more sensitive to others: “I’ve become more caring, I care more about people, because of my mother’s illness.” Another, who had lived with an SIP for more than 10 years, highlighted feeling strong and persistent: “I’ve become very good at never giving up. I try again, even if things are hard, always.” She attributed this strength to her values: she felt strong because she valued relationships and “the small things in life”, rather than expensive phones and clothes. Another participant emphasized that she had to get stronger, in order to not feel weakened by the parental illness: “You have to try and rid yourself of the weakness, or make yourself stronger, or try to improve so that you don’t get so weak, or sad anymore.” Personal development could also mean being more conscious about how much family meant to her: “I think a lot about how much I love my family, and how much I appreciate the time we have together. I’m not sure I would have thought the same if my family hadn’t been in this situation.”

Some had become more independent and helpful at home because of expanded domestic responsibilities. One boy, who lived alone with his ill father, explained that chemotherapy significantly reduced his father’s physical capacity. He had spoken to his mother about the situation and, in the interview, related his response to her expressed concern about his coping: “I said ‘Me? I will manage.’” With a proud and confident voice, he then stated, “And I did.”

### Relational aftershocks—gains and losses

In addition to the personal strain, the participants’ accounts illustrated that parental somatic illness could mean relational strain, both within the family and among friends. These descriptions of relational strain can be interpreted and conceptualized as relational aftershocks. The contrasting descriptions of valued changes in the families’ highlight that parental somatic illness can mean gains as well as losses. Notably, participants also reported that family life continued like “normal”.

#### Welded family ties—and formation of cracks

Several of the participants described feeling grateful, as the parental illness had increased family closeness. One applied the metaphor of *welding* to explain what happened when both parents became ill: “She fell ill, and then he fell ill. Then we became even more welded together as a family.” The participant continued by describing, whilst smiling, how the parental illnesses strengthened the love between her parents, and how it had brought her closer to her ill father as he reoriented his life after the onset of his illness. Part of this reorientation involved prioritizing hobbies, which resulted in more time spent together. She explained:

When dad got sick, he was like, ‘You only live once’. He always thought he would prioritize his hobbies when he retired, but now he has realized that it can all be over before you know it. And that you have to fulfil your dreams.

Other participants highlighted fewer quarrels as the reason for increased family closeness. One participant said, “My brother and I have been fighting less. I feel that we’ve got a better relationship since we’ve been through exactly the same thing.”

To some participants, the parental illness meant increased distance in the family; interpreted as formation of cracks. A girl, who portrayed herself as optimistic and calm, avoided her younger sister, and described her as pessimistic and dramatically emotional. The participant explained why she avoided her sister: “I find her annoying, because she cries eeeeevery evening. I get so tired of it, you wouldn’t believe it.” Another participant wondered if his parents’ recent divorce and subsequent reduced contact between family members occurred because of the parental illness. Accounts of how ill parents were unable to participate in family activities—for example, meals or trips—also illustrated cracks in family life. At the same time, it is noteworthy that several participants stated that many aspects of their family life, such as relationships, daily routines and communication patterns, were “normal” and unchanged despite parental illness. As emphasized by one participant, whose ill fathers’ cancer treatment seemed successful: “It has not changed the family in any way.”

#### Challenging times for friendships

Participants reported that having a somatically ill parent was mentally exhausting because it challenged their relationships with their friends. Some participants described frequent, uncomfortable situations in which friends pitied them, cried or expressed shock after being updated on the parent’s illness. One girl emphasized the burden it was to update friends about her ill parent’s situation, as they expressed surprise every time she did so. She used dramatic intonation when imitating her friends’ response during the interview: “Seriously!!? Are you okay????!” She explained that such explicitly dramatic reactions were burdens, as they scared her. In addition, more subtle reactions were also challenging, like this one described by a participant who had new friends visiting to prepare for a party:

Dad came into the kitchen, and by then he was bald. He’s not fond of wearing a wig, so he walks around bald everywhere. Then everyone sort of got quiet. I said, ‘As you probably understand, my dad has cancer’ and then everyone sort of got quiet.

This participant did not expect her ill father to enter the room, nor her friends’ reaction. The uncomfortable silence in the kitchen was accompanied by insecurity regarding what to say. The participant explained that she had divergent needs—she wanted them to continue their conversation, but she also wanted her friends to show they cared. In the end, she resolved the situation: “They probably didn’t know what to say. I had to say it was okay, and that I preferred talking about something else.” She thus had to dissolve the relational insecurity in addition to leaving her need for care behind.

Two participants highlighted the impact of relational aftershocks related to friendship by saying that, instead of their family situation, it was their friends and school-related matters that determined how they felt. One participant illustrated this in her description of how, though her expanded domestic responsibilities were difficult and time-consuming, the most stressful aspect was her friends’ reactions:

I think housework can be fun, sometimes. However, my friends do not understand why I cannot see them, if I have promised to clean the windows. They do not understand, and maybe they think I do not want to meet them. *That*’s been very difficult; it’s been hard. I’ve tried to explain, but they don’t understand.

Thus, what she actually found problematic about her expanded domestic responsibilities was her friends’ lack of understanding with regards to her situation.

Participants also often referred to situations where friends began treating them differently than before, or differently than they treated other friends. One participant explained how, when she was calm and quiet, friends would ascribe her tranquillity to the parental illness—an overgeneralization that she found frustrating. In contrast, no one reacted if other schoolmates were calm and quiet. One participant, who had just began at a new school, did not inform her new schoolmates about her ill parent to avoid being perceived as, “the one with the ill mother”. Another used a metaphor that emphasized vulnerability: “For me, it was important that they didn’t treat me any differently, as if I were made of glass.” She disliked being seen solely as vulnerable, sad and suffering.

## Discussion

Participant’s narratives constituted a rich source of knowledge regarding how parental somatic illness influence adolescents’ mental health. Following Antonovsky (1987), parental somatic illness represents a stressor life event followed by chains of events that produce tension in several areas of the lives of adolescents with an SIP.

The discussion that follows emphasizes the results related to parental somatic illness as a challenging time for adolescents’ friendships, as this has received little attention in earlier studies. Notably, this result confirms the significance of friendships during adolescence, a period in which independence- and identity-seeking constitute central developmental tasks (Erikson, 1963; P. K. Smith, 2016). Next, results related to adolescents’ occupied minds, personal development and perceptions of aftershocks in the family will be discussed.

For study participants, parental somatic illness led to two types of mental strain related to friendships. Dealing with their friends’ immediate reactions when participants talked about their situation was difficult. Correspondingly, a recent study (Tozer et al., 2019) found that, for adolescents, talking about parental cancer was difficult, due to people’s reactions and because such conversations sometimes meant talking about intimate parts of the body. The present study highlights how adolescents with an SIP might experience the moment in which friends learn about the parental illness as difficult—such as in the example mentioned above, when a participant’s bald father suddenly entered the kitchen, leading to what the participant experienced as an uncomfortable situation, as her friends did not know what to say. The participant resolved the situation by stating she preferred to talk about something else, even though she had actually wanted her friends to show that they cared. This example also illustrates additional phenomena. First, unexpected situations in which the parental illness is exposed might be uncomfortable for adolescents; notably, in the above example, the decision to reveal the parental illness was not *hers*. Thus, the example also illustrates the importance of communication about *when and how* to include adolescents’ friends in the story about the parental illness. As the father walked in when his daughter had new friends visiting, preparing for a party, the situation also illustrates the importance of parental sensitivity regarding what openness about the illness means in different contexts. Perhaps a carefree evening was more important than openness that evening? However, perhaps the dad *was* conscious of the implications of his entering—he may have believed it would result in increased support for his daughter. Finally, this example illustrates a strain-inducing situation in which the participant had to choose between meeting her own needs for care and her friends’ needs to escape an uncomfortable situation. Hence, it was a dilemma of whether to prioritize her own needs or the perceived needs of her friends. This was a common trait across the ethical dilemmas described by other participants. Adolescents’ dilemmas around whose needs to cover (and when) have also been reported in earlier studies, in which adolescents with an SIP described being torn between the desire to spend time with friends or study abroad and the desire to stay home to support the ill parent (Bogosian et al., 2011; Mauseth & Hjälmhult, 2016). It has also been found that other adolescents with an SIP avoid initiating wished-for conversations so as not to upset parents (Kennedy & Lloyd-Williams, 2009b).

Some participants described a long-lasting mental strain from the perception that friends treated and saw them differently after being informed about the parental illness. Understanding identity as the interactions between one’s self-perception and others’ perceptions of one’s self (Nelson, 2002), this mental strain can be considered identity-related. At its core is the participants’ perception that their friends have a poor understanding of what life with an SIP means: in particular the presence and absence of mental strain, personal growth and increased relational closeness in the family. Participants described how many of their friends seemed to presume a constant presence of mental strain due to the parental illness; as such, there are divergent narratives. In order to conceptualize “the lens” through which adolescents with an SIP describe being perceived, we developed the concept of a “tristopticon” (derived from Latin, *tristis*, meaning “sad”) from “Malopticon”. Malopticon is a construction of subjects as untrustworthy (McNeill, 2018)—a metaphorical apparatus or process through which a person is seen as “bad”. Correspondingly, tristopticon is a metaphorical apparatus or process through which a person is seen solely as “sad”. As one of adolescents’ developmental tasks is to detach from the family and acquire independence through shaping their own identity (P. K. Smith, 2016), the identity-related strain of being seen through the tristopticon might be experienced as especially challenging for adolescents with an SIP, compared to those who are younger or older. The burden of being perceived through the tristopticon, as “the adolescent with an SIP”, highlights the relevance of the emphasis placed by salutogenesis and person-centeredness on perceiving all persons as unique individuals rather than as patients (McCormack & McCance, 2017; Vinje et al., 2017).

One of the examples presented above suggests that it might be difficult to escape the tristopticon: recall the participant who hesitantly expressed that she felt better as her father’s illness grew more serious, and her repeated emphasis that this must sound “strange” and “weird”. Her hesitations and repetitions might be interpreted as a fear of being perceived as insensitive to her father’s situation. Accordingly, she experienced a dilemma in which expressing her feelings represented a risk of being misunderstood. Alternatively, her hesitations and repetitions might mean that the idea of coexisting states of happiness and sadness was unfamiliar to her. This interpretation highlights the relevance of understanding mental health as a dynamic movement along a continuum (Antonovsky, 1979). It might therefore be helpful for adolescents with an SIP to learn about this approach to mental health, as it underlines how one might experience both happiness and sadness in the face of parental somatic illness, and that there can be variations due to different situational contexts.

The adolescents’ descriptions of troubled thoughts and feelings due to parental illness are supported by earlier studies (Boström & Nilsagård, 2016; Phillips & Lewis, 2015), and their descriptions of situations in which they were unsure of what to do—i.e., of the ethical dilemmas they experienced—add to our knowledge of possible mental strain following parental illness. These experiences illustrate that life with a somatic illness might lead to challenged norms for interaction, as it constitutes an unusual and unknown situation in which people are unsure of what to do (Hauken & Dyregrov, 2015).

The participants’ descriptions of personal development and of increased family closeness due to the parental illness are consistent with other studies’ results (Cipolletta & Amicucci, 2015; Jansson & Anderzen-Carlsson, 2017; Kissil et al., 2010). Following Antonovsky (1979, 1987), the adolescents’ personal development and increased family closeness may be due to their access to sufficient resources to resolve the tensions created by parental illness. One participant applied the metaphor of being “welded together” to express increased family closeness due to parental illness. The metaphor symbolically underlines the relevance of the super-ordinate theme of living in an earthquake zone; both earthquakes and welding imply the potential to create new structures. Still, our results also show that parental illness might increase relational distance in the family, a result consistent with earlier studies (Cipolletta & Amicucci, 2015; Tozer et al., 2019). Notably, participants’ accounts suggest that a sibling who reacts to the situation quite differently can constitute a mental strain.

Some of the participants said that the parental illness had not severely influenced their mental health or aspects of their family life. Here, an emphasis on taking what participants said at face value might lead to the inference that their lives were unmarked by the illness. Still, the participants’ descriptions of the mental strains they experienced challenge this interpretation. An alternative interpretation could be that participants were reluctant to talk about painful experiences during the interview, or that they actively focused on those aspects of their mental health and family life that they experienced as stabile. While these interpretations might be valid, it is important to be aware that they undermine adolescents’ chance to voice their experiences of “normalcy” and to escape the tristopticon. An interpretation in line with Antonovsky’s (1979, 1985) understanding of mental health as a dynamic movement along the ease–dis/ease continuum could be that adolescents’ lives, at the time of the interview, *were* good, but that life with an SIP means frequent movement along the continuum. This interpretation is capable of containing both experiences of untroubled joy as well as worries and sadness. Next, mental health as a frequent movement along the continuum is supported by earlier IPA research describing mental health as constantly continuous, ongoing movement—“like walking up and down a staircase” (Mjøsund et al., 2015, p. 215). Regardless, these complexities emphasize the importance of interpreting statements concerning what mental health or family life is like in the face of parental somatic illness with care.

### Implications for practice and further research

Our study indicates that mental health promoting efforts towards the mental health of adolescents with an SIP need to be directed towards both personal and relational strains. In addition to supporting adolescents with an SIP with their troubling thoughts and feelings, it seems crucial to understand their ethical dilemmas concerning whose needs to cover (and when). Supporting families is also key in promoting unifying processes—when seeking to understand the mental strains experienced by adolescents with an SIP, it therefore seems important to include questions of how other family members and friends react to the situation. Finally, adolescents with an SIP could be supported in their process of increasing control over and improving their own health (WHO, 1986), through reflecting on which mental strains of parental somatic illness they experience, and which resources they have found effective in resolving that strain.

Future research is needed concerning what adolescents with an SIP perceive as useful resistant resources (Antonovsky, 1979, 1987) when faced with the mental strains created by parental somatic illness. A deeper understanding of how adolescents with an SIP perceive and solve the ethical dilemmas would likely also prove relevant. Finally, it seems imperative that adolescents with an SIP be given the chance to voice their experiences of “normalcy” and good mental health in future research.

### Strengths and limitations

We consider the main strength of the study to be its in-depth focus on the perceptions of adolescents with an SIP concerning the mental strain of parental illness—a focus that enabled us to recognize that parental illness represents a particularly challenging period with regards to the adolescents’ friendships.

One possible limitation is that adolescents who struggled intensely with their family situation may have avoided participating in the study. Moreover, through their positions as gatekeepers during recruitment, health-care personnel and parents might have neglected to inform adolescents in especially vulnerable situations about the study.

## Conclusion

Adolescents with an SIP perceived that parental somatic illness means both personal and relational strain and growth. Results support earlier results, as Norwegian adolescents in this study seem to experience parental somatic illness similarly to adolescents from other countries. Moreover, study results nuance the existing knowledge of parental somatic illness as a challenging time for adolescents, particularly by highlighting the under-recognized relational strains experienced when being with friends. Accordingly, the recognition of this interdependency needs increased awareness. The results underline the significance of health personnel paying attention to both personal and relational strains when developing and providing information and follow-up for adolescents with an SIP. At the same time, we suggest that the results might contribute in promoting parents and other significant others in their efforts to understand and support adolescents with an SIP.

## Acronyms

General resistant resources (GRRs)

Interpretative, phenomenological analysis (IPA)

Somatically ill parent (SIP)
